# Assessment of apolipoprotein B/apolipoprotein A-I ratio in non-ST segment elevation acute coronary syndrome patients

**DOI:** 10.1186/s43044-020-00057-1

**Published:** 2020-05-24

**Authors:** Haitham Galal, Ayman Samir, Mohamed Shehata

**Affiliations:** Department of Cardiology, Faculty of Medicine, Ain Shams University, Cairo, Egypt

**Keywords:** Myocardial infarction, Coronary angiography, Lipoproteins

## Abstract

**Background:**

The apolipoprotein B/apolipoprotein A-I ratio was shown to be strongly related to the risk of myocardial infarction in several large-scale studies. The current study aimed at exploring the diagnostic and short-term prognostic values of apolipoprotein B/apolipoprotein A-I ratio in patients presenting with non-ST segment elevation acute coronary syndrome. One hundred patients with non-ST segment elevation acute coronary syndrome were prospectively enrolled, in addition to a matched group of 100 patients with chronic stable angina. Serum levels of total cholesterol, low-density lipoprotein, high-density lipoprotein, triglycerides, and apolipoproteins B and A-I were quantified in both groups. Patients with non-ST segment elevation acute coronary syndrome underwent coronary angiography.

**Results:**

The mean age of the study population was 57 ± 6 years, 65% being males. The non-ST segment elevation acute coronary syndrome group showed significantly unfavorable lipid profile parameters, including apolipoprotein B/apolipoprotein A-I ratio. Higher apolipoprotein B/apolipoprotein A-I ratio was associated with more coronaries showing significant stenosis and more complex lesion morphology. Receiver operating characteristic curve analysis reached an optimal cut-off value of 0.93 for diagnosis of non-ST segment elevation acute coronary syndrome (sensitivity 70% and specificity 88%) and 0.82 for predicting the presence of multi-vessel disease (sensitivity 90% and specificity 97%).

**Conclusion:**

Apolipoprotein B/apolipoprotein A-I ratio is a useful tool of risk assessment in patients presenting with non-ST segment elevation acute coronary syndrome including prediction of coronary multivessel affection.

Apolipoprotein B/apolipoprotein A-I ratio was shown to be strongly related to risk of myocardial infarction. Higher ratios of apolipoprotein B/apolipoprotein A-I were recorded in NSTE-ACS patients (versus stable angina patients). Higher apolipoprotein B/apolipoprotein A-I ratios were associated with more diseased coronaries and complex lesions. Apolipoprotein B/apolipoprotein A-I ratio is a useful tool for acute risk assessment in cardiac ischemic patients.

## Background

Apolipoproteins are proteins linked with lipids within lipoprotein particles. They are known to play essential roles in lipoprotein metabolism. They transfer these hydrophobic molecules in aqueous media of plasma directing lipids to their target organs, activating or inhibiting enzymes implicated in lipid metabolism [1]. Apolipoprotein A-I (apoA-I) is the largest ingredient of the high-density lipoprotein cholesterol (HDL-C) particle, constituting about 45% of its molecular mass [2]. Apolipoprotein B (apoB) exists in chylomicrons, e.g., apoB-48, and in very low-density lipoproteins cholesterol (VLDL-C), intermediate density lipoprotein cholesterol, and low-density lipoprotein cholesterol (LDL-C) [3–5]. In several large-scale studies, the apoB/apoA-I ratio was related strongly to the risk of development of myocardial infarction and other cardiovascular diseases. Accumulating data suggest that apoB/apoA-I ratio could be a predictor of acute coronary syndromes in addition to the well-known elevated LDL-C and plasma total cholesterol/high-density lipoprotein cholesterol ratio as strong predictors of cardiovascular disease [6–9]. Direct assessment of the concentration of apoB was assumed to be superior in assessment of atherogenic dyslipidemia [10], and it is worth to mention that laboratory assessment of apolipoproteins is not affected by fasting status [11].

In the current study, the authors sought to determine the role of apoB/apoA-I ratio in risk assessment of development of non-ST segment elevation acute coronary syndrome (NSTE-ACS) in patients presented with acute typical chest pain. We also explored the short-term prognostic value of apoB/apoA-I ratio in NSTE-ACS patients meant for coronary angiography and subsequent revascularization.

## Methods

### Study design and data collection

A total of 100 patients presented with NSTE-ACS were enrolled on prospective basis. They were admitted to the coronary care unit (CCU) within the first 6 h of onset of chest pain, in the period between December 2016 and February 2018. All the included patients met the clinical criteria of NSTE-ACS (acute coronary syndromes without ST segment elevation including unstable angina (UA) and non-ST segment elevation myocardial infarction (NSTEMI). NSTEMI is defined [12] by the rise and fall of cardiac biomarkers (preferably troponin) with at least one value above the 99th percentile upper reference limit and accompanied by one of the following: anginal chest pain, new ST segment/T-wave changes, development of pathologic Q waves on ECG, or imaging evidence of new regional wall motion abnormality. UA is defined by the presence of clinical symptoms of cardiac ischemia (new-onset typical chest pain, or change in typical anginal pattern, or development of angina at rest), with normal cardiac biomarkers of injury (troponin). ST segment depression or T-wave inversions may be present in ECG.

Electrocardiographic assessment was performed on admission. A matched group of 100 patients with chronic stable angina was prospectively enrolled. The following patients were excluded: patients with prior history of acute coronary syndrome (ACS), with prior percutaneous coronary intervention, or coronary artery bypass graft surgery; patients with any myocardial disease other than ischemic; and those with history of lipid-lowering drug use (in NSTE-ACS group) or those on high intensity statins (for stable angina patients). Patients with contraindications for aspirin and/or clopidogrel use, limited life expectancy due to coexistent morbidities (e.g., malignancy), and chronic liver or kidney disease (serum creatinine ≥ 1.5 mg/dl or alanine aminotransferase and aspartate aminotransferase of ≥ 2 times normal) were also excluded. Before enrollment, informed written consent was acquired from all patients and the study protocol was ratified by our local institutional human research committee, as it complies to the ethical guidelines of the Declaration of Helsinki quoted in 1975, as revised in 2008.

### Echocardiographic assessment

Assessment of both segmental and global left ventricular systolic functions was performed by trans-thoracic echocardiography employing a GE Vivid 7 cardiac ultrasonic machine (GE, Horten, Norway). Standard 2D, M-mode, and Doppler images were acquired using a 2.5-MHz phased array probe. Modified Simpson`s method was used to assess left ventricular (LV) ejection fraction. LV internal dimensions using M-mode and wall motion abnormalities were recorded. The standard 17-segment model was used to assess segmental wall motion abnormalities as applied by the American Society of Echocardiography [13]. Each segment was scored according to the grading system described by Knudsen et al. [14]. Views were acquired and analyzed upon CCU admission (in the NSTE-ACS group) by a single echocardiographer, who was blinded to the study protocol. Another blinded echocardiographer analyzed the views acquired for the chronic stable angina group.

### Laboratory work-up

Venous sampling for cardiac troponin I was taken upon CCU admission for patients belonging to the NSTE-ACS group. Patients with initial negative cardiac troponin I results were subjected to serial measurements (every 12 h) for 48 h. Plasma concentration of cardiac troponin I was measured using the Troponin I Ultra assay by Siemens ADVIA Centaur system (Siemens Diagnostics, Germany). Detection value was 6 pg/mL, with a 99th percentile of 40 pg/mL, and a coefficient of variation of < 10% at 30 pg/mL. The serum levels of total cholesterol, LDL-C, HDL-C, and triglycerides (TG) were measured using standard methods. Fasting samples were withdrawn for chronic stable angina patients, while samples from the NSTE-ACS patients were withdrawn upon CCU admission (within 6 h of onset of chest pain). Serum apolipoprotein B and A-I levels were recorded using latex agglutination assays (Daiichi Pure Chemicals, Japan). The non-HDL-C concentration was calculated as the total cholesterol minus the HDL-C.

### Coronary angiography

Patients belonging to the NSTE-ACS group underwent coronary angiography within the first 48 h after CCU admission. Vascular access was obtained through femoral artery puncture in all patients. Standard angiographic views were obtained. Subsequently, coronary revascularization strategies were tailored individually. Coronary angiographic data were interpreted by 2 independent operators, blinded to the study protocol. It included the number of vessels (epicardial vessels of ≥ 2.5 mm diameter) exhibiting significant luminal stenosis (≥ 70% diameter reduction), maximum grading of luminal stenosis (percentage of diameter reduction), and worst morphologic lesion type (type A, B, or C) defined according to the American College of Cardiology/American Heart Association (ACC/AHA) angiographic classification of coronary lesions [15].

### Statistical analysis

The sample size was projected from the study of Krintus et al. [16], which evaluated the use of apoB/apoA-I ratio in order to enhance the process of assessment of the risk of ACS. Power of the test was set to 80%. The confidence interval was set to be 95%, and the margin of error was set to 5%. Sample size was quoted to be 100 patients per group. All continuous variables were described as mean ± standard deviation (± SD). Categorical variables were described using absolute and relative (percentage) frequencies. Comparisons of continuous variables between the study groups were done using Student’s *t* test. For categorical data, Pearson’s chi-square test was performed. Kappa (*k*) statistics with the confidence interval was used to assess inter-rater reliability (*k* was 0.95). ANOVA test was used to estimate the relationship between apoB/apoA-I ratio and each of the number of significantly diseased coronaries and worst atherosclerotic lesion type. *P* value was used to describe significance (*P* ≤ 0.05 is significant value, *P* ≤ 0.01 is considered highly significant value, and *P* ≥ 0.05 is a non-significant result). ROC curve (receiver operating characteristic curve) was used to obtain the optimal cut-off value of ApoB/ApoA-I ratio in order to predict the presence of significant ≥ 2 vessel disease in NSTE-ACS patients. Statistical calculations were done employing the Statistical Package for Social Sciences (SPSS for Windows) software (version 15.0, SPSS Inc., Chicago, USA).

## Results

### Baseline characteristics

A total of 200 patients were subjected to the study protocol. The mean age of the whole study cohort was 57 ± 6 years, 130 (65%) being male patients. Both study groups were matched regarding age, gender, and risk factor of coronary artery disease. Forty-five (45%) patients among the NSTE-ACS group were finally diagnosed as non-ST segment elevation myocardial infarction. There was no recorded significant difference between both study groups regarding echocardiographic data, body mass index, and pre-enrollment medications (Table 1).

**Table 1:** Baseline characteristics of the two study groups

Variable	NSTE-ACS (no. = 100)	CSA (no. = 100)	*P* value*
Age,(years)	59 ± 6	58 ± 5	0.601
Males	66 (66)	64 (64)	0.598
Hypertension	55 (55)	51 (51)	0.544
Diabetes mellitus	30 (30)	32 (32)	0.598
Dyslipidemia	58 (58)	56 (56)	0.568
Smoking	37 (37)	40 (40)	0.696
Family history of CAD	17 (17)	18 (18)	0.589
Body mass index (kg/m^2)^	25 ± 2	26 ± 3	0.654
Left ventricle ejection fraction (%)	51 ± 5	54 ± 3	0.543
Left ventricle end diastolic diameter (mm)	56 ± 4	53 ± 4	0.435
Left ventricle end systolic diameter (mm)	39 ± 3	37 ± 5	0.556
Wall motion score index	1.45 ± 0.3	1.42 ± 0.22	0.476
Pre-enrollment medications			
Beta blockers	12 (20)	14 (24)	0.589
Calcium antagonists	11 (18)	9 (16)	0.597
Angiotensin converting enzyme inhibitors/angiotensin receptor blockers	18 (30)	17 (29)	0.644
Oral nitrates	13 (22)	12 (21)	0.688

Categorical variables are presented as number (percentage)

Continuous variables are presented as mean ± standard deviation

*CAD* coronary artery disease, *CSA* chronic stable angina, *NSTE-ACS* non-ST segment elevation acute coronary syndrome

*Pearson’s chi-square and Student’s *t* tests

### Blood work

Data analysis showed that the NSTE-ACS group exhibited significantly higher total cholesterol, LDL-C, non-HDL-C, TG, total cholesterol/HDL-C, LDL-C/HDL-C, TG/HDL-C, and apoB and apoB/apoA-I values. However, the chronic stable angina group showed significantly higher HDL-C and apoA-I values (Table 2).

**Table 2:** Lipid profile indices of the two study groups

Variable	NSTE-ACS (no. = 100)	CSA (no. = 100)	*P* value*
Total cholesterol (mg/dl)	195 ± 31.2	183.3 ± 31.2	**0.025**
Serum LDL cholesterol (mg/dl)	124.8 ± 19.5	105.3 ± 23.4	**0.03**
Serum HDL cholesterol (mg/dl)	39 ± 15.6	50.7 ± 11.7	**0.03**
Non-HDL-C (mg/dl)	156 ± 7.8	132.6 ± 19.5	**0.002**
Serum triglycerides (mg/dl)	115.7 ± 26.7	89 ± 17.8	**0.002**
Total cholesterol/HDL cholesterol	5 ± 2	4 ± 1.6	**0.003**
Triglycerides/HDL cholesterol	1.3 ± 0.8	0.8 ± 0.7	**0.0006**
LDL cholesterol/HDL cholesterol	3.2 ± 1.3	2 ± 1.5	**0.0005**
Apolipoprotein B (g/L)	1.6 ± 0.3	1 ± 0.02	**0.002**
Apolipoprotein A-I (g/L)	0.9 ± 0.2	1.3 ± 0.2	**0.002**
Apolipoprotein B/apolipoprotein A-I	1.7 ± 0.2	0.7 ± 0.1	**0.0002**

All variables are presented as mean ± standard deviation

*HDL* high-density lipoprotein, *LDL* low-density lipoprotein

*Student’s *t* test

Among the NSTE-ACS patients, apoB/apoA-I ratio showed a significant positive correlation with total cholesterol (*r* = 0.477, *P* = 0.016), LDL-C (*r* = 0.596, *P* = 0.002), non-HDL-C (*r* = 0.577, *P* = 0.003), total cholesterol/HDL-C (*r* = 0.389, *P* = 0.025), and LDL-C/HDL-C (*r* = 0.554, *P* = 0.004). ApoB/apoA-I ratio among chronic stable angina patients showed weaker (less significant) positive correlations with the same parameters as follows: total cholesterol (*r* = 0.357, *P* = 0.045), LDL-C (*r* = 0.457, *P* = 0.015), non-HDL-C (*r* = 0.389, *P* = 0.025), total cholesterol/HDL-C (*r* = 0.377, *P* = 0.035), and LDL-C/HDL-C (*r* = 0.457, *P* = 0.015). ApoB/apoA-I ratio did not show any statistically significant correlation with age, echocardiographic parameters, HDL-C, TG, and TG/HDL-C, in both study groups.

### Coronary angiography

Only 85 (85%) patients (NSTE-ACS group) showed angiographically significant coronary artery disease. Angiographic data were interpreted by 2 interventional cardiologists, who were not informed about the study protocol. Analysis of inter-observer variability revealed a close correlation between repeated interpretations, with a correlation coefficient of *r* = 0.95.

Patients in the NSTE-ACS group were sub-classified according to number of coronary vessels (one vessel, two vessels, and > 2 vessels) showing significant stenosis. Using ANOVA test, it was found that there was a significant difference between the three sub-populations regarding mean apoB/apoA-I ratio (*P* = 0.02). Higher mean apoB/apoA-I ratio was significantly associated with more coronary arteries affection. Patients were re-classified according to frequency of presence of worst type of coronary lesions (lesion type A, B, or C). Using ANOVA test, it was found that there was a significant difference between the three sub-populations regarding mean apoB/apoA-I ratio (*P* = 0.03). Higher mean apoB/apoA-I ratio was significantly associated with more complex lesion morphology (Table 3), and this was justified by a post hoc analysis test revealing that any group with higher apoB/A-I ratio was more significant than the other with lower apoB/A-I ratio. Patients were further re-classified according to maximum degree of luminal stenosis (70 to < 80%, 80 to < 90%, 90 to < 100%, 100%). There was no statistically significant correlation between recorded apoB/apoA-I ratio and degree of angiographically determined maximum luminal stenosis (Pearson’s correlation coefficient, 0.154; *P* = 0.07) (Table 4).

**Table 3:** Mean ApoB/Apo A-I ratio in relation to the number of significantly diseased coronaries and type of coronary lesions in NSTE-ACS group

Sub-population		Number of patients^†^	Apolipoprotein B/apolipoprotein A-I^‡^	F*	*P* value*
Number of significantly diseased vessels	1	53 (53)	1.6 ± 0.02	737.7	**0.02**
	2	20 (20)	1.8 ± 0.04		
	> 2	12 (12)	1.9 ± 0.04		
Type of worst lesion	A	48 (48)	1.6 ± 0.04	3.8	**0.03**
	B	23 (23)	1.8 ± 0.07		
	C	14 (14)	1.9 ± 0.07		

*ANOVA test

^**†**^Variables are presented as number (percentage)

^**‡**^Variables are presented as mean ± standard deviation (SD)

**Table 4:** Mean ApoB/ApoA-I ratio in relation to maximum coronary luminal stenosis in the NSTE-ACS group

Sub-population		Number of patients*	Apolipoprotein B/apolipoprotein A-I^†^	*P* value	
Percentage of luminal stenosis (%)	70 to < 80	24 (34)	1.7 ± 0.03	**0.7** (*r* = 0.154)	
	80 to < 90	36 (36)	1.8 ± 0.04		
	90 to < 100	18 (18)	1.8 ± 0.06		
	100	7 (7)	1.7 ± 0.05		

*r* Pearson’s correlation coefficient

*Variables are presented as number (percentage)

**†**Variables are presented as mean ± standard deviation (SD)

**Fig. 1:**
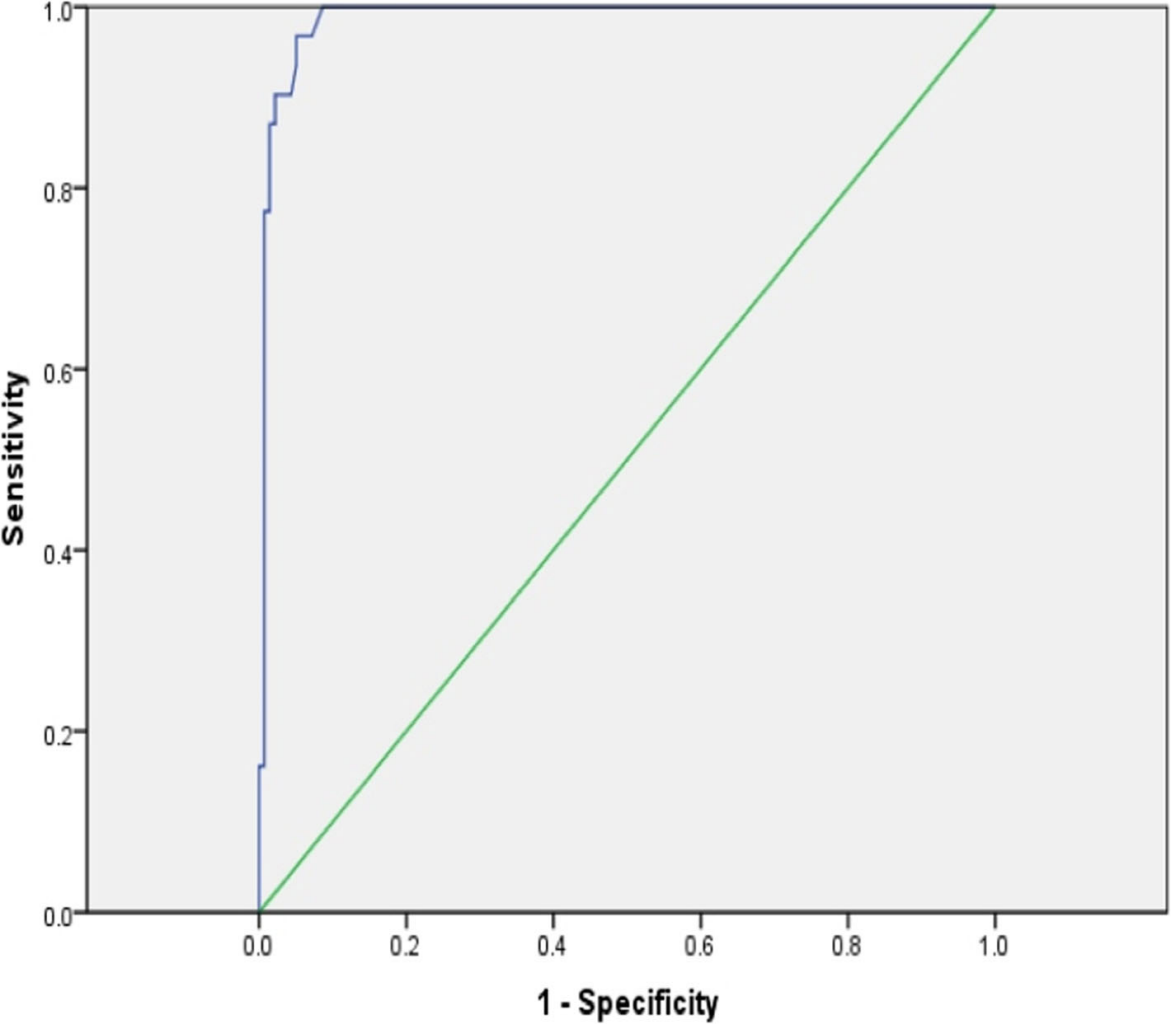
Receiver operating characteristic (ROC) curve plotted to obtain the optimal cut-off value of ApoB/ApoA-I ratio in order to predict the presence of significant ≥ 2 vessel disease in NSTE-ACS patients

Receiver operating characteristic curve analysis (Fig. 1) of apoB/apoA-I ratios among the NSTE-ACS patients showing significant coronary artery disease revealed that a cut-off value of ≥ 0.82 predicted the presence of ≥ 2 vessel disease, with sensitivity of 90%, specificity of 97%, positive predictive value of 88%, negative predictive value of 99%, and predictive accuracy of 98% (area under the curve = 0.97, 95% CI 0.782–0.973). It may be worth to mention that the receiver operating characteristic curve analysis reached an optimal cut-off value of 0.93 for diagnosis of non-ST segment elevation acute coronary syndrome (sensitivity 70% and specificity 88%), and of course, this needs a large-scale study population to reveal the proper diagnostic ability of the apoB/A ratio in the NSTE-ACS population.

## Discussion

The current study presents 2 different roles for apoB/apoA-I ratio in NSTE-ACS patients. The first one can be described as a tool of risk assessment of development of NSTE-ACS in patients presenting with acute typical chest pain. The other one presents a short-term prognostic tool in NSTE-ACS patients undergoing coronary angiography in terms of multivessel affection and lesion morphology which subsequently could affect the revascularization outcome. One of the well-known risk factors in ischemic patients is elevated level of LDL-C. Despite several studies indicating the necessity for LDL-C recording in patients at risk of ACS development, centering only on LDL-C is not suggested as an ideal diagnostic and/or therapeutic approach [17]. The coexistence of high TG and low HDL-C, called atherogenic dyslipidemia, is linked to high apoB concentration, and as the role of apolipoproteins in coronary risk estimation is still debatable, the apoB/apoA-I ratio is considered a rising star predictor factor [18]. The results of both AMORIS and INTERHEART studies showed that the apoB/apoA-I ratio was the most powerful predictor of myocardial infarction among all investigated parameters and importantly, like C-reactive protein, was able to detect subjects at higher risk even when LDL-C values were normal [5, 8]. In the current study, patients presented with NSTE-ACS showed unfavorable conventional lipid profile parameters, as compared with chronic stable angina patients. Moreover, they showed higher apoB and apoB/apoA-I ratio. These significant differences were recorded although both groups of included patients showed a comparable risk factor profile and were statin naïve. This could be solely attributed to the pathophysiologic nature of ACSs, including the inflammatory milieu. The authors sought to highlight the role of apoB/apoA-I ratio in risk assessment by estimating a cut-off value of 0.93, predicting the diagnosis of NSTE-ACS. Previous trials had reported that an apoB/apoA-I ratio of ≥ 0.9 is associated with increased risk of myocardial infarction [5, 8]. This cut-off value was obtained through follow-up of patients with considerable risk of coronary artery disease. The current study reported the same cut-off value. However, blood samples were withdrawn at time of presentation to CCU (within 6 h of acute chest pain), giving apoB/apoA-I ratio an additional diagnostic impact and confirming the results obtained by previous studies. Considering the cut-off point of this reliably measured ratio could provide a promising short-term prognostic tool in this clinical setting, when compared with conventional lipid profile parameters. Apolipoproteins are measured directly through accurate and internationally validated methods [19], by using a common reference method for apoA-I and apoB which is not obtainable for measurements of HDL-C and LDL-C, and without the interference of high TG levels [20]. Plasma apolipoprotein levels are mildly affected by biological variables, whereas plasma lipid levels vary in response to different metabolic factors [4]. Therefore, the measurements of apolipoproteins B and A-I can be done with no need of prior fasting [7, 21]. These facts add more to the advantageous assessment of apoB/apoA-I ratio.

If the total apoB reflects the possibly atherogenic lipoproteins and apoA-I transports the major anti-atherogenic HDL particles, the apoB/apoA-I ratio could reasonably provide a measure of the gross cholesterol balance [22]. That was evident in the current study through the positive correlation between apoB/apoA-I ratio and each of total cholesterol, LDL-C, non-HDL-C, total cholesterol/HDL-C, and LDL-C/HDL-C, which was more pronounced in NSTE-ACS patients. While some studies showed the usefulness of increased apoB levels as predictors of cardiac risk [23, 24], others have attributed this risk to reduced apoA-I levels [25]. However, the consensus in the literature showed that the ratio between atherogenic and anti-atherogenic particles, reflected by the apoB/apoA-I ratio, represents an additional and important parameter for cardiovascular risk prediction [26]. We excluded patients on statin therapy to avoid confounding factors. Multiple studies involving the apoB/apoA-I ratio revealed that lipid-lowering drugs, especially statins, have significant effects on the apolipoproteins levels [27–30].

The present study uniquely reported the relationship between apoB/apoA-I ratio and coronary angiographic features in NSTE-ACS patients. Higher apoB/apoA-I ratios were associated with more coronary arteries showing angiographically significant coronary artery disease and more lesion complexity. However, there was no significant correlation between apoB/apoA-I ratio and degree of luminal stenosis. We propose that NSTE-ACS is a setting of enhanced oxidative stress and elevated lipoprotein levels, conferring a pronounced pro-inflammatory thrombogenic milieu. On the other hand, a study by Tsimikas et al. reported a positive correlation between apolipoproteins and degree of coronary luminal stenosis [31]. They showed that plasma levels of oxidized phospholipids present on apoB-100-containing lipoproteins reflect the presence and extent of angiographically documented coronary artery disease. However, their study was conducted on stable ischemic patients.

The results of the current study have their assumed clinical implications. High apoB/apoA-I ratio (≥ 0.82) recorded before coronary angiography in NSTE-ACS patients calls for proper readiness (pharmacological and technical) due to high probability of tackling a complex multi-vessel disease. This additionally implies the presence of an experienced interventionalist. Thus, apoB/apoA-I ratio could help risk stratification of NSTE-ACS patients upon CCU admission. These patients are expected to have unfavorable angiographic outcomes, in spite of initial and/or persistent negative troponin results. The current study reached a cut-off value of 0.93 for apoB/apoA-I ratio that predicted the final diagnosis of NSTE-ACS. This could be of special importance in patients with initial negative troponin results. This population of patients should be regarded as a high-risk category among ACS patients. Moreover, this could have an impact on pharmacological treatment in CCU.

### Limitations of the study

The concluded data presented in this study only apply for patients designated by inclusion and exclusion standards. This is a single-center study with a relatively small sample size. Serial sampling for apoB/apoA-I ratio, especially after coronary revascularization, was not included in the study protocol. Furthermore, follow-up of adverse cardiovascular events was also not included. Further multi-center studies with wider scope are needed for evaluation of long-term impact of high apoB/apoA-I ratio in revascularized NSTE-ACS patients.

## Conclusions

ApoB/apoA-I ratio is a useful tool for risk assessment in patients presenting with acute typical chest pain. High apoB/apoA-I ratio predicts multi-vessel (cut-off value of 0.82) coronary artery disease with complex lesion morphology.

## Data Availability

The datasets used and analyzed during the current study are available from the corresponding author on reasonable request.

## Abbreviations

NSTE-ACS: Non-ST segment elevation acute coronary syndrome
ApoB/ApoA: Apolipoprotein B/apolipoprotein A
HDL-C: High-density lipoprotein cholesterol
LDL-C: Low-density lipoprotein cholesterol
CCU: Coronary care unit
ACC/AHA: American College of Cardiology/American Heart Association
